# October

**Published:** 1890-10-11

**Authors:** 


### OCTOBER.

OCTOBER was the eighth month of the old Roman year, but by the Julian arrangement it became the tenth month, and had thirty-one days assigned to it. By the Slavs it was called the "yellow month," from the fading of the leaf—emblematic of man, "now green in youth, now withering in the ground." To the Anglo-Saxons it was known as "winter-fylleth," because at the October full moon winter was esteemed to have commenced. The sun is now getting low, and the last days of October, although joyous to the Indian, as the "Ras Jatra," and amongst some northern peoples sacred to fire, was nevertheless a season of fear and trembling. For the last days of October usher in November, the windy "blot month," or "bloody month." Nevertheless, October has joys and beauties of its own, for the month is "crowned with the sickle and the wheaten sheaf." At the beginning of the month the trees have already assumed the glorious autumnal tints, preparatory to the general fall which will be easily accomplished before the "Blot month" sets in. Often enough, the weather is wet in October, but on the other hand there are frequently glorious October days, when the sun shines with a brilliancy suggestive of a much more southern climate. From the average of fifty years' meteorological observations, it is found that there is a gradual reduction of mean temperature from the first to the last day of October, the figures being for the two periods, 54 deg. and 47 deg. F. It is true that in October we do not yet begin to feel the real cold of winter. But we are sensible of the change of temperature if out in the evening, when "condensed descend the exhalations of the cool declining year"; or if abroad in the morning when moisture sparkles on the filmy gossamer of the field spider, of which Buffon calculated 663,552 webs to make a single pound! Wise persons will therefore make some change of clothing, especially if they remember the old saying, that a cough caught in October endures throughout the winter. Neither is there lack of choice, for now purveyors of clothing present to an enduring public all kinds of complicated arrangements in the way of capes, coats, and cloaks "for those in whose knees rheumatism is wont to settle," as one advertisement has it. The *Field* tells us that "with changeable weather and variable temperature the time has now arrived for taking cattle into a yard." Similarly, the time has now arrived when many think of warmer climates. Let, however, invalids consider well before giving up the comforts of home for the benefit of the climate of the south. No doubt it is a great matter to escape the fogs, damp, rain, and winds of an English November, and of an English winter generally. And the contrast between a damp, foggy, London suburb, or even a British sea-side resort, and the Riviera with its brilliant sun, azure sea, and wealth of flowers, is great. But the Riviera is not reached without a somewhat fatiguing and expensive journey. Then there are cold winds on the Riviera, emphasised by the warm and brilliant sun, so that passing even from the sunshine to the shade, demands for the invalid an extra wrap. Again, after sunset, an immediate and great fall of temperature takes place. Sanitary arrangements may not be good, although owing to the complaints of English sojourners, such arrangements have been generally recently much improved. If a person has comparatively little the matter, only requiring change and escape from the fog and damp, by all means let him go to the Riviera about the end of this month of October, if he can afford it! But if he be really ill, do not let him attempt the journey, especially if he cannot afford to have, not only everything that is necessary, but also all that is desirable when there. Fortunately, there are a large number who do not desire to go abroad, and who young, strong, and healthy, even prefer the British winter. Many such, think October-on-Tweed the essence of enjoyment. We are rather inclined to agree with those who regard angling as chiefly consisting in waiting for a bite, during which the *piscator* hums or whistles softly, "Tommy make room for your uncle," or some other monotonous tune. Waiting on the bank of a river, or in a punt, or, still worse, wading, are very certain methods of getting a cold or a rheumatism, especially if assisted by the "ventilated water-proof, but not air-proof" garment. We opine that for any length of time, or during any exertion, the only waterproof allowable is the boot and a very short cape on the shoulders. But the commencement of hunting also demands attention. Hunting of one kind or another has always been a favourite sport of Englishmen, as the following old lines show:

> "Give me the naked heaven above
> The broad bare heath below,
> A merry glance from her I love,
> My fleet hound, and my bow!"

As we are not ruled by Ireland, probably hunting will not become extinct at present. One person advertises how to clean faded hunting coats, which is a very laudible aspiration; but another advertises waterproof garments for hunting, which we cannot approve of. A waterproof may be carried elsewhere, according to the taste of the rider, but not on his back, until it rains. It appears necessary for comfort, although we do not know that it has much to do with health, that hunters should wear breeches of a particular shape. "It will seem odd," says one firm of breeches makers, "to persons whose legs are usually covered with trousers, that so much skill should be required for cutting a well-fitting pair of breeches." And another firm say, gentlemen desirous of avoiding a journey to London will find themselves well suited with breeches at Maidstone. We imagine, however, that most hunting men visit London a little before the hunting season, and perhaps it would be well if they took a few lessons on the immediate treatment of accidents and injuries, so that they might be in a position to not, at least, maltreat those accidents and injuries, so frequently happening in the hunting-field. Another manner in which persons often come to grief in October is by attendance at coursing meetings and autumnal race meetings. They stand about in the cold, they get a chill, and when they return home they suffer from a cold in the head, if fortunate, otherwise from rheumatism, lumbago, bronchitis, or pneumonia. Ought not persons, therefore, to go to such meetings in October? Of course they may go, but then they should take more care of themselves than they usually do, and remember that although the pitcher goes to the well and returns whole very often, it gets broken at last. When after the summer heat, "staid autumn walks with rustling feet;" when the zodiacal sign typifies the turning of the balance from heat to cold, from light to darkness, it is necessary for those who would preserve their health to make corresponding change of clothing. We have not space for all we could say about this month of October. Otherwise, we might tell of going a-nutting in the days when we were young with ——— but it does not matter! We might mention how the hazel leaves turning yellow hid the bushy-tailed squirrel, indignant at encroachment on his nut preserves. We might tell of the dormouse preparing for the winter sleep, of the last feathered lingerers collected for their flight to more sunny lands, and of the first winter visitors, the woodcock, the snipe, the red-wing, all, perhaps, from lands where "sport" is unknown, ignorantly exposing themselves to the attacks of civilization. October 12th is the anniversary of the publication of James I.'s Dæmonology," wherein it was stated, "In a secret murder, if the dead carkasse be at any time handled by the murderer it will gush out of blood, as if the blood were crying for revenge." This was a popular article of belief, not only in this country, but in foreign lands also. October 14th, 1643, was the date when Basing House, Hants, was taken by Cromwell. The Holy Ghost Chapel was then stripped of lead for the purpose of making bullets for the besiegers. On October 19th, 1741, Garrick, the British Roscius, first appeared at the theatre in Goodman's Fields. Small bills, still preserved, show that the prices of admission were 3s., 2s., and 1s., while the modern irritating practice of charging for programmes had not been invented. October 21st is the anniversary of the battle of Trafalgar in 1805. England's fleet consisted of 27 sail of the line and four frigates. The combined fleets of France and Spain numbered 23 sail of the line and seven frigates. The battle lasted four hours, terminating in a British victory, but saddened by the death of Nelson. October 24th was the date when hawking commenced at the period when this sport was a fashionable amusement. Then there was the "Great Falconer," one of the principal officers of State. The flight of the hawk after the quarry was thus described: "He carries lightning in his wings, he strikes the trembling bird." On October 26th an old book advises every one to be blooded, a custom once very prevalent at the rise and fall of the year, but now honoured by breach, and not observance. October 29th, old style, was Lord Mayor's Day, when at the Lord Mayor's dinner, "the carver dancing round each dish, surveyed with flying knife" the meats he was soon to supply to the Aldermen. It would appear that the accomplishment of carving was esteemed of greater importance in former times than now. Thus Burton says of a lady: "She is fit to bear the office (of a wife), govern a family, bring up children, sit at boards and carve." And in the "History of Chivalry" we find the following:—

"Now when the Lord he did come home
For to sit down and eat,
He called for his daughter dear
To come and carve his meat."

We scarcely think the girl of the period would respond if called for such service.

